# Fatal and extensive multiorgan hemorrhages in anti-melanoma differentiation-associated gene 5 antibody-positive dermatomyositis: An autopsy case report

**DOI:** 10.1097/MD.0000000000018600

**Published:** 2020-01-17

**Authors:** Tsuyoshi Watanabe, Naoho Takizawa, Toru Nagasaka, Yoshihiro Nakamura, Hiroki Ikai, Mari Yamamoto, Yukari Murai, Koji Takasugi, Waka Yokoyama-Kokuryo, Yoshiro Fujita

**Affiliations:** a Department of Rheumatology; b Department of Pathology; c Department of Nephrology, Chubu Rosai Hospital, Minato-ku, Nagoya, Aichi, Japan.

**Keywords:** anti-MDA5 antibody, diffuse alveolar damage, multiorgan hemorrhages, rapidly progressive interstitial lung disease

## Abstract

**Introduction:**

Anti-melanoma differentiation-associated gene 5 antibody (anti-MDA5 Ab) is an autoantigen associated with dermatomyositis (DM). Anti-MDA5 Ab-positive DM patients frequently exhibit clinically amyopathic dermatomyositis (CADM), and develop rapidly progressive interstitial lung disease (RPILD). Even with early detection and potent combination immunosuppressive therapy, anti-MDA5 Ab-positive DM patients have a poor prognosis. In the present case report, we present a rare autopsy case of a patient with anti-MDA5 Ab^+^ DM with RPILD who exhibited diffuse alveolar damage (DAD) patterning in lung specimens, and extensive hemorrhages in multiple organs.

**Patient concerns:**

An 82-year-old Japanese man admitted with bacterial pneumonia was subsequently diagnosed with anti-MDA5 Ab-positive DM based on skin manifestations (mechanic's hand, ulcerated palmar papules, and flagellate erythema), myositis, interstitial pneumonia, and elevation of anti-MDA5 Ab titer.

**Diagnosis:**

The patient was diagnosed with anti-MDA5 Ab^+^ DM, complicated with RPILD.

**Interventions:**

The patient received potent immunosuppressive therapy consisting of pulse methylpredonisolone at a dose of 1000 mg for 3 days, followed by prednisolone at 60 mg/d, a 1000 mg pulse of intravenous cyclophosphamide (IVCY), and oral tacrolimus at 6 mg/d. Intravenous immunoglobulin (IVIG) at a dose of 400 mg/kg/d for 5 days was subsequently administered.

**Outcomes:**

Despite triple immunosuppressive therapy and IVIG, the patients’ respiratory status deteriorated, and the patient died of respiratory failure on the twelfth day after admission. An autopsy revealed pulmonary DAD and multiorgan hemorrhages, including the left iliopsoas muscle, gastric and bowl mucosa, spleen, and left adrenal gland.

**Lessons:**

Multiorgan hemorrhages may be a fatal complication in anti-MDA5 Ab^+^ DM patients.

## 1 Introduction

Dermatomyositis (DM) is an autoimmune inflammatory myopathy with characteristic cutaneous involvement. Accumulating evidence suggests that several myositis-specific autoantibodies are associated with specific clinical features, such as distinctive skin lesions, muscle involvement, interstitial lung disease (ILD), underlying malignancy, and mortality. Anti-melanoma differentiation-associated gene 5 antibody (anti-MDA5 Ab) was identified as an autoantigen associated with DM in 2009.^[1]^ Anti-MDA5 Ab-positive (^+^) DM patients frequently exhibit clinically amyopathic dermatomyositis (CADM) and develop rapidly progressive interstitial lung disease (RPILD). Although a strategy of early detection and potent combination immunosuppressive therapy (high-dose glucocorticoid, cyclosporine, and intravenous cyclophosphamide pulse) can improve the survival rate of anti-MDA5 Ab^+^ DM patients, the mortality rate remains high at 25% even with triple therapy.^[2]^

The causes of death in anti-MDA5 Ab^+^ DM patients are significantly associated with respiratory failure via RPILD,^[3,4]^ and lung specimens at autopsy exhibit histopathological diffuse alveolar damage (DAD).^[5]^ However, further investigation is needed to understand the pathological conditions associated with deterioration in anti-MDA5 Ab^+^ DM patients with RPILD. In this case report, we present a rare autopsy case of an anti-MDA5 Ab^+^ DM patient with RPILD who presented with DAD in his autopsy lung specimen, and extensive hemorrhages in multiple organs.

## 2 Case report

**Figure 1:**
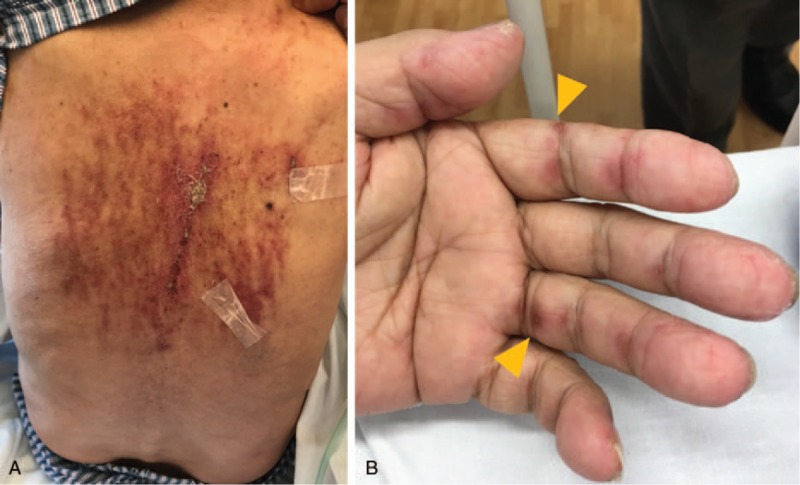
Clinical image of flagellate erythema; widespread linear scratch-like lesions with erythema on the back on admission day (A). Palmar papules on palms and fingers (B).

An 82-year-old Japanese man presented at the emergency department with 4 weeks of generalized weakness and 2 days of dyspnea. His medical history included hypertension and prostate cancer, which had been treated by radical prostatectomy 5 years earlier. He was taking losartan and amlodipine for the hypertension. An initial physical examination revealed bibasilar coarse crackles in both lungs, and scratch-like erythematous skin lesions on his back (Fig. 1A). On admission, his blood pressure, heart rate, respiratory rate, and oxygen saturation were 88/65 mmHg, 110 beats per minute, 22 breaths per minute, and 92% in ambient air, respectively. He was afebrile with a temperature of 36.3 °C. He had no signs of paralysis. Manual muscle testing revealed mild weakness of the iliopsoas and quadriceps muscles.

**Figure 2:**
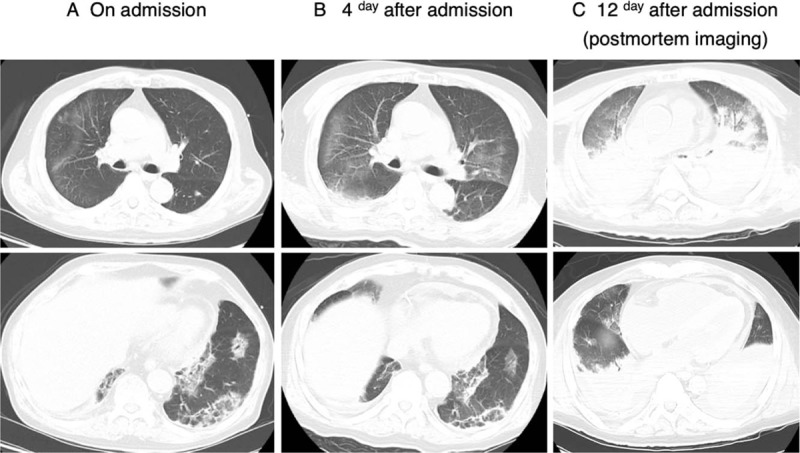
Chest CT on admission showing the presence of peripheral consolidation in the left lower lung base and ground-glass opacities in lung fields (A). Ground-glass opacities exacerbated in the bilateral lung fields, without improvement of peripheral consolidation in the left lower lung base (B). Postmortem chest CT examination revealed widespread consolidations in both lung fields and dramatic loss of lung volume (C).

Laboratory evaluation revealed a white blood cell count (WBC) of 8900 cells/μL, hemoglobin (Hb) of 11.3 g/dL, and normal platelet counts (PLT). The level of creatine kinase (CK) was elevated at 924 U/L (normal range, 124–222 ng/mL). The levels of C-reactive protein (CRP), lactate dehydrogenase (LDH), aspartate aminotransferase, and alanine aminotransferase were elevated at 5.6 mg/dL, 682 U/L (normal range, 124–222 U/L), 305 U/L, and 180 U/L, respectively. Kidney dysfunction was also detected (creatinine [Cr] at 2.81 mg/dL, blood urea nitrogen [BUN] at 74.0 mg/dL). Chest radiography revealed multiple small pulmonary infiltrates in both lungs and additional high-resolution computed tomography (HRCT) of the chest revealed the presence of peripheral consolidation in the left lower lung base, and ground-glass opacities in all 6 lung fields (Fig. 2). Expectorated sputum was not good quality, and the sputum culture results were unremarkable; however, a pneumococcal urinary antigen test was positive. The patient was initially diagnosed as having community-acquired pneumonia and severe sepsis, and was subsequently started on fluid replacement and antibiotic treatment with ceftriaxone and azithromycin.

After antibiotic and fluid replacement treatments, the patient's blood pressure and kidney function improved, but his symptoms of dyspnea and weakness worsened on the fourth day after admission. Physical examination revealed fine crackles at the lung base. The patient also developed slight skin thickening on the lateral side of the fingers (consistent with mechanic's hand) and ulcerated palmar papules (Fig. 1B) in addition to already-existing multiple vertical linear streaks with spotty crusting on his back (flagellate erythema). A chest HRCT scan revealed exacerbation of the ground-glass opacities in the bilateral lung fields, without improvement of peripheral consolidation in the left lower lung base (Fig. 2B). A bronchoscopy was performed to examine the possibility of an infectious cause, but no noteworthy colonies were isolated in bacterial or mycobacterial cultures from the bronchoalveolar lavage sample.

**Figure 3:**
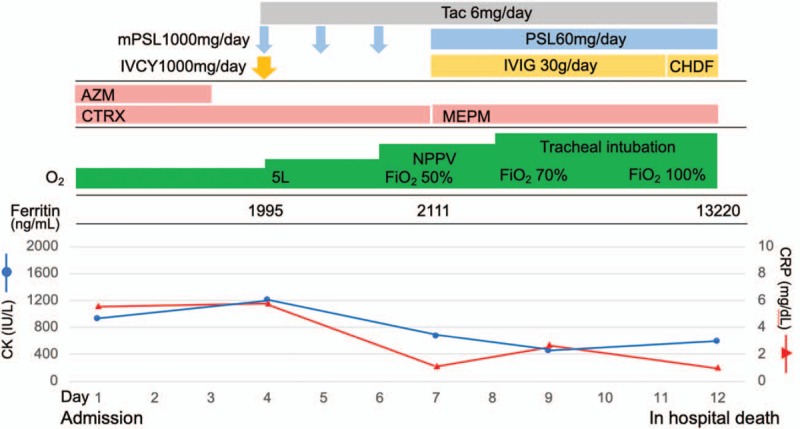
Clinical course of the patient. AZM = azithromycin, CHDF = continuous hemodiafiltration, CK = creatine phosphokinase, CRP = C-reactive protein, CTRX = ceftriaxone, IVCY = intravenous cyclophosphamide, IVIG = intravenous immunoglobulin, MEPM = meropenem, mPSL = methylprednisolone, NPPV = noninvasive positive pressure ventilation, Tac = tacrolimus.

Based on the clinical suspicion of acute interstitial pneumonia due to DM, high doses of methylprednisolone pulses (1 g/d for 3 days), 1000 mg of intravenous cyclophosphamide (IVCY), and oral tacrolimus (6 mg/d) were administered on the fourth day after admission (Fig. 3). The laboratory data showed the following results: white blood cell count (WBC 7400 cells/μL, Hb 14.3 g/dL, PLT 11.4 × 10^4^/μL, LDH 728 U/L, CK 1208 U/L, CRP 5.78 mg/dL, and KL-6 1146 U/mL). The patient's ferritin level was also elevated at 1995 ng/mL (normal range, 30–300 ng/mL). Following immunological and serological testing, anti-MDA5 Ab was found to have a high titer index (>150; normal range, 0–39). Anti-aminoacyl tRNA synthetase (anti-ARS) antibody, anti-transcription intermediary factor 1-gamma (TIF1-γ) antibody, anti-Mi-2 antibody, antinuclear antibody (ANA), proteinase 3-anti-neutrophil cytoplasmic autoantibodies (PR3-ANCAs), and myeloperoxidase anti-neutrophil cytoplasmic autoantibodies (MPO-ANCAs) were all negative. The patient was eventually diagnosed as having anti-MDA5 Ab-positive DM with RPILD. The patient received intravenous immunoglobulin (IVIG) in addition to triple immunosuppressive therapy. However, his respiratory status exacerbated, and this was complicated by upper gastrointestinal bleeding without apparent coagulation abnormality on the ninth day after admission. Although the patient received intensive treatment with mechanical ventilatory support, continuous infusion of vasopressors, and continuous hemodiafiltration, he died of respiratory failure on the twelfth day after admission. The postmortem CT examination revealed widespread consolidations in both lung fields and dramatic loss of lung volume (Fig. 2C).

**Figure 4:**
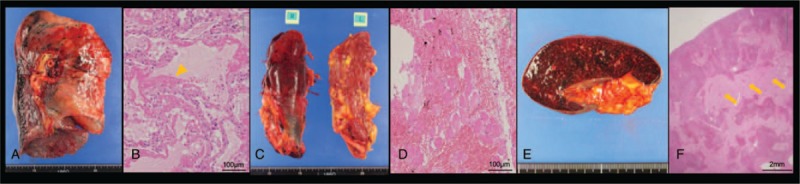
Autopsy findings of lung specimens (A) were consistent with DAD, with findings of hyaline membrane formation, and fibrotic tissue covering the alveoli (arrowhead) (B, Hematoxylin and eosin stain, Bar 100 μm). A specimen obtained from the right iliopsoas muscle (C) showed intramuscular bleeding; however, a specimen from the quadriceps muscle showed no definitive inflammation (D, Hematoxylin and eosin stain, Bar 100 μm). In the spleen specimen (E), extensive intraparenchymal hemorrhages were observed (arrow) (F, Hematoxylin and eosin stain, Bar 2 mm).

An autopsy was performed 2 hours after death. Both lungs revealed a congestive increase in weight (745 g for the left and 760 g for the right) with sporadic alveolar hemorrhages (Fig. 4A). Autopsy specimens taken from the lungs were consistent with DAD, with findings of hyaline membrane formation and fibrotic tissue covering the alveoli (Fig. 4B). A specimen taken from the right iliopsoas muscle showed intramuscular bleeding (Fig. 4C); however, a specimen from the quadriceps muscle showed no definitive presence of inflammatory cells (Fig. 4D). In addition, extensive intraparenchymal hemorrhages were observed, including the gastric and bowl mucosa, spleen (Fig. 4E and F), and left adrenal gland.

## 3 Discussion

Anti-MDA5 Ab^+^ DM patients frequently develop RPILD. Due to the severity and rapid progression of the disease, the prognosis of anti-MDA5 Ab^+^ DM complicated by RPILD is poor, with a 90-day survival rate of 67% in Asia.^[6]^ Although early diagnosis and treatment of anti-MDA5 Ab^+^ DM are important to improve patient outcomes, it is difficult to distinguish rapidly deteriorating abnormalities such as dyspnea, weakness, elevation of muscle enzymes, and pulmonary involvement associated with anti-MDA5 Ab^+^ DM from those caused by severe bacterial pneumonia.

For early recognition and treatment of anti-MDA5 Ab^+^ DM, careful attention should be focused on the unique cutaneous manifestations, which might reflect severe underlying vasculopathy. The characteristic cutaneous manifestations include cutaneous ulcers, alopecia, lateral digit hyperkeratosis or scaling, palmar papules, oral ulcers, and panniculitis.^[7]^ In the present case, the patient had initially developed widespread linear, scratch-like lesions with erythema on his back, which was consistent with flagellate erythema. Although flagellate erythema represents an active disease state in only 5% of patients with DM, its prevalence in anti-MDA5 Ab^+^ DM patients is unknown.^[8]^ In the initial evaluation of the patient in the current case, the linear streaks were not considered to be cutaneous lesions caused by DM. After admission, palmar papules and ulcer-like lesions were observed on the patient's hands, and consequently, the combination of those cutaneous manifestations and antibiotic therapy-refractory RPILD raised the probability of DM complicated with ILD, especially anti-MDA5 Ab^+^ DM.

We initiated potent immunosuppressive therapy on the fourth day after admission, before positive confirmation of anti-MDA5 Ab, and immediately after failure of antibiotic therapy and recognition of the distinctive skin manifestations of DM. This potent immunosuppressive therapy consisted of high-dose glucocorticoids, an IVCY pulse, and oral calcineurin inhibitors, and was previously shown to improve the survival rate of anti-MDA5 Ab^+^ DM patients.^[2]^ However, approximately 25% of anti-MDA5 Ab^+^ DM patients did not survive even with triple therapy. In one study, a serum ferritin level of >1000 ng/mL before therapy, ground-glass opacities in all 6 lung fields before therapy, and worsening pulmonary infiltrates during therapy were identified as poor prognostic indicators, and patients with all 3 factors usually died despite receiving triple immunosuppressive therapy.^[9]^

The autopsy examination revealed acute and organized DAD, and invasion of macrophages into the alveoli. Although DAD is consistent with the clinical state called adult respiratory distress syndrome (ARDS), which can be caused by infection, malignancy, highly concentrated oxygen, shock, trauma, drugs, and radiation, there was no evidence of infection, malignancy, or other causes in this patient. In previous reports, 7 anti-MDA5 Ab^+^ DM patients, including the present case, were histopathologically examined by autopsy,^[5,10–14]^ and lung specimens were consistent with DAD in 6 of the 7 cases. In the present case, distinctive extensive hemorrhages were also observed within the alveolae, iliopsoas muscle, gastric mucosa, spleen, and adrenal gland. We reviewed other known cases concerning anti-MDA5 Ab^+^ DM patients, and found only 2 previously published cases with organ bleeding.^[9,12]^ In one case, the patient developed pneumomediastinum and intramuscular bleeding,^[9]^ whereas in another case, diffuse alveolar damage and alveolar hemorrhage were observed by autopsy.^[12]^ However, the mechanism responsible for organ hemorrhages in anti-MDA5 Ab+ DM cases was not determined in these previous studies. Multiorgan hemorrhages might be a rare but fatal complication in anti-MDA5 Ab^+^ DM patients, or might be an elusive complication, since hemorrhages were not detected before the autopsy in our case. The evidence of disseminated intravascular coagulation, which might cause multiorgan hemorrhages due to coagulation abnormality, was absent until the day before death. Therefore, further investigation would clarify the association between the anti-MDA5 Ab and extensive hemorrhages in multiple organs. We speculate that DAD caused by anti-MDA5 Ab^+^ DM was the major cause of death, and concurrent multiorgan hemorrhages may have affected the deteriorating condition of the patient.

In conclusion, anti-MDA5 Ab^+^ DM with RPILD is associated with poor prognosis even with the administration of early aggressive immunosuppressive agents. Multiorgan hemorrhages might be a rare but fatal complication in patients with anti-MDA5 Ab^+^ DM with RPILD.

## Acknowledgments

The authors would like to acknowledge the members of the Department of Rheumatology at the Chubu Rosai Hospital, Japan.

## Author contributions

**Conceptualization:** Tsuyoshi Watanabe.

**Investigation:** Tsuyoshi Watanabe.

**Project administration:** Tsuyoshi Watanabe.

**Supervision:** Naoho Takizawa, Toru Nagasaka, Yoshihiro Nakamura, Hiroki Ikai, Mari Yamamoto, Yukari Murai, Koji Takasugi, Waka Yokoyama-Kokuryo, Yoshiro Fujita.

**Writing – original draft:** Tsuyoshi Watanabe.

**Writing – review & editing:** Tsuyoshi Watanabe, Yoshiro Fujita.

Tsuyoshi Watanabe orcid: 0000-0002-5189-9584.
